# Hypothalamic tanycytes generate acute hyperphagia through activation of the arcuate neuronal network

**DOI:** 10.1073/pnas.1919887117

**Published:** 2020-06-08

**Authors:** Matei Bolborea, Eric Pollatzek, Heather Benford, Tamara Sotelo-Hitschfeld, Nicholas Dale

**Affiliations:** ^a^School of Life Sciences, University of Warwick, Coventry CV4 7AL, United Kingdom

**Keywords:** tanycyte, hypothalamus, food intake, arcuate nucleus, ATP

## Abstract

Hypothalamic tanycytes are chemosensitive glial cells that contact the cerebrospinal fluid in the third ventricle and send processes into the hypothalamic parenchyma. To test whether they can activate neurons of the arcuate nucleus, we targeted expression of a Ca^2+^-permeable channelrhodopsin (CatCh) specifically to tanycytes. Activation of tanycytes ex vivo depolarized orexigenic (neuropeptide Y/agouti-related protein; NPY/AgRP) and anorexigenic (proopiomelanocortin; POMC) neurons via an ATP-dependent mechanism. In vivo, activation of tanycytes triggered acute hyperphagia only in the fed state during the inactive phase of the light–dark cycle.

## Significance

Tanycytes are nutrient-sensing cells that line the third ventricle within the hypothalamus. The role of tanycytes in the regulation of food intake has not been documented. Indeed, the mechanistic link between nutrient concentrations in the CSF and activation of neurons responsible for the regulation of food intake, such as orexigenic (NPY/AgRP) or anorexigenic (POMC) cells, is not yet clear. Here, we demonstrate that tanycytes, engineered to express channelrhodopsin, can activate arcuate neurons to induce acute hyperphagia when activated by light. These data provide further evidence that tanycytes are an integral link between CSF nutrients and the hypothalamic neuronal networks that regulate appetite and energy balance.

Alteration of hormonal levels and abnormal circulating nutrients is linked to obesity and diabetes. Food intake is regulated centrally, specifically by neural networks in the hypothalamus and brainstem. Within the hypothalamus, the arcuate (ARC) nucleus, the dorsomedial hypothalamic nucleus, and the paraventricular nucleus interact closely to control energy balance—the net outcome of regulating food intake, energy storage, and energy expenditure. Arcuate neurons which express agouti-related protein (AgRP) and neuropeptide Y (NPY) are activators of food intake during a metabolic deficit. By contrast, arcuate neurons that express proopiomelanocortin (POMC) are activated during excessive food intake and are regarded as anorexigenic. Both neuronal populations interact together to fine-tune food intake and control energy balance (1–3). In addition to their role in the homeostatic control of body weight via integration of information encoded by circulating nutrients and hormones, both AgRP/NPY and POMC neurons can exhibit rapid responses to sensory cues that influence food intake (4–6).

So far, investigations have concentrated mainly on the neuronal mechanisms underlying the control of body weight (7, 8). However, the role of glial cells has recently become a focus of interest (9–11). There has been particular interest in hypothalamic tanycytes, a specialized type of glial cell, that line the third ventricle. Their cell bodies contact the cerebrospinal fluid (CSF) and send a single process into the hypothalamic parenchyma reaching nuclei such as the ARC and the ventromedial hypothalamic nuclei (12, 13). Historically, these cells have been organized in four subtypes dorsally to ventrally (α_1_, α_2_, β_1_, β_2_) according to their anatomical position and morphological characterization. However, very little is known about their anteroposterior organization (14). Anatomically, therefore, tanycytes lie at the center of the neuronal circuits that control body weight, leading to speculation that they could contribute to the functioning of these circuits. At least some of these cells have been described as potential neural stem cells and give rise to new neurons within the arcuate nucleus postnatally (14). Tanycytes are able to transport leptin and ghrelin across the blood–brain barrier, thus enabling the central action of these hormones on the hypothalamic neurons (15–17). Similarly, thyroid hormones are transported and activated by tanycytes (18).

Tanycytes are also chemosensitive: α_2_ and β_1_ tanycytes can sense nutrients such as glucose, amino acids, and fatty acids in the CSF. These stimuli can trigger Ca^2+^ signals and local release of ATP that acts on tanycytes and close cells via purinergic receptors (19–23). In addition to this, β_2_ tanycytes release FGF21 in response to circulating free fatty acids to regulate lipolysis in peripheral fat stores (24–27). An increasing body of evidence therefore supports the hypothesis that tanycytes convey information on peripheral nutrient levels to the effector neurons in the hypothalamic network to alter energy balance. However, direct evidence for these cells communicating with the central primary effectors of feeding (arcuate AgRP/NPY and POMC neurons) to influence food intake is lacking.

We have addressed this question by targeting expression of a Ca^2+^-permeable version of channelrhodopsin2 (CatCh) via a cell-specific promoter to tanycytes to permit their specific activation by light. We have made whole-cell recordings from neurons of the ARC and find that tanycytes can activate neurons in the ARC, which include NPY- and POMC-expressing neurons. To further understand the role of tanycytes in the hypothalamic neuronal network controlling food intake, we studied the effect of tanycyte activation during the inactive (light) phase of the light–dark cycle and following a 19-h period of fasting.

## Results

### Dynamics of Tanycyte Ca^2+^ Signaling.

**Fig. 1.**
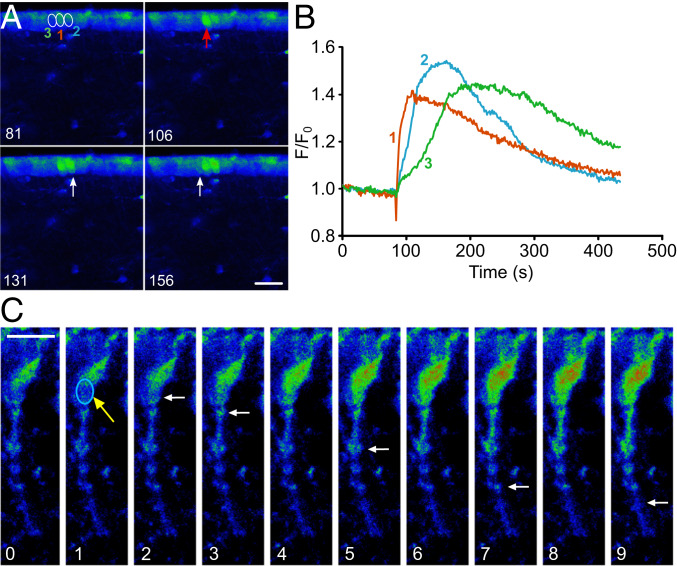
Ca^2+^ signaling evoked by infrared stimulation of a single tanycyte. (*A*) Montage showing images of the Ca^2+^ response (Rhod-2) to infrared stimulation of single tanycyte (ROI 1 and red arrow). The tanycytes on either side (ROIs 2 and 3 and white arrows) exhibit an increase of Ca^2+^ following the stimulation of tanycyte 1. The numbers in the lower left corners represent the times of the images (s) and match the graph in *B*. (Scale bar, 20 µm.) (*B*) Quantification of the change in Ca^2+^ illustrated in *A*. Note that tanycyte 3 reaches its peak response some 100 s after the initial stimulation of tanycyte 1. (*C*) Montage showing a single tanycyte. The soma was stimulated (blue ROI and yellow arrow) and a localized increase in Ca^2+^ was induced, which then traveled along the process (white arrows). Numbers in lower left corners represent the times of the images in seconds. (Scale bar, 10 µm.)

To test the effect of activating just a single tanycyte, we loaded tanycytes with Rhod-2 AM and exploited the fact that a brief exposure to infrared illumination (720 to 730 nm) within a region of interest (ROI) smaller than the soma can evoke a localized elevation of intracellular Ca^2+^ in that ROI (Fig. 1, *SI Appendix*, Fig. S1, and Movies S1–S3). While this has not been previously reported, we observed this Ca^2+^ mobilization in 50 out of 117 tanycytes tested. The elevation of intracellular Ca^2+^ appeared to arise, at least partially, as a result of release from intracellular stores. It was observed in 65 out of 221 cells recorded when extracellular Ca^2+^ had been substituted by Mg^2+^ and 1 mM ethylene glycol bis(2-aminoethyl)tetraacetic acid (EGTA) was present to chelate any remaining extracellular Ca^2+^. The infrared stimulation was repeatable, as a second stimulation in zero Ca^2+^ saline could evoke an additional Ca^2+^ wave in 21 out of 41 tanycytes. In addition, there were no detectable changes in morphology following stimulation, suggesting that it was not damaging the tanycytes (Fig. 1, *SI Appendix*, Fig. S1, and Movies S1–S3). Infrared stimulation of tanycytes is thus a convenient tool to cause highly localized mobilization of Ca^2+^ from intracellular stores in subregions of tanycytes that can be precisely defined by laser scanning.

Infrared stimulation of an ROI within a single tanycyte was sufficient to evoke a Ca^2+^ signal in that tanycyte and that could then spread to neighboring tanycytes (Fig. 1 *A* and *B*, *SI Appendix*, Fig. S1, and Movies S1–S3). A wave of Ca^2+^ activation in the tanycyte layer could propagate to as many as eight neighboring cells in both the dorsal and ventral directions. While our slices were cut in the coronal plane, we have previously shown that Ca^2+^ waves travel through the tanycyte population anteroposteriorly too, in parasagittal hypothalamic slices (21). On average, the wave traveled 13.8 ± 1.2 µm (*n* = 84)—this is equivalent to a mean distance of about two tanycyte cell bodies. The mean speed at which a tanycyte induced activation of neighboring tanycyte spread was 1.2 ± 0.2 µm⋅s^−1^ (±SEM; *n* = 84).

By utilizing infrared stimulation of ROIs in single tanycyte somata, we could follow the progress of the Ca^2+^ wave as it traveled down the tanycyte process away from the stimulated ROI (Fig. 1*C*). This wave traveled at a mean speed of 2.4 ± 0.5 µm⋅s^−1^ (±SEM; *n* = 13). We observed that Ca^2+^ signals could propagate out of the field of view of the microscope (110 × 110 µm), suggesting that they could propagate along the entire length of the process (see also Movies S1–S3; cf. supplementary movies in ref. 21). Thus, stimulation of a single tanycyte will cause a wave of activation in neighboring tanycytes and a signal that propagates into the hypothalamic parenchyma. These two features of tanycyte signaling make it plausible that tanycytes could activate the hypothalamic neuronal networks, albeit with dynamics limited by the slow speed of Ca^2+^ signaling.

**Fig. 2.**
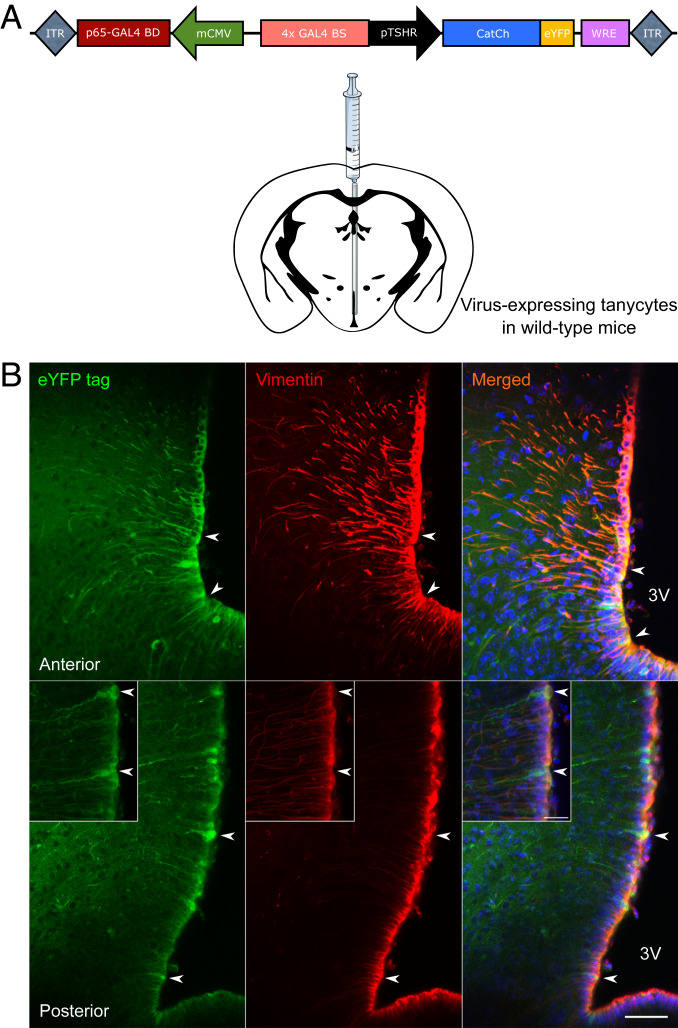
Experimental design and transduction of tanycytes with AdV-pTSHR-CatCh. (*A*) Schematic of the viral construct used to be able to have a specific expression of CatCh in tanycytes (AdV-pTSHR-CatCh). A similar control was injected that only contained a GFP-based protein (21). The bidirectional construct uses the strong CMV promoter to drive transcription/translation of a p65-GAL4 fusion protein. This fusion protein then binds to the GAL-4 binding site (BS) adjacent to the TSHR promoter, where the p65 moiety enhances transcription via the cell-specific TSHR promoter, thereby enhancing the expression of the CatCh-eYFP protein. (*B*) Immunohistochemistry of hypothalamic sections expressing the viral vector (AdV-pTSHR-CatCh; an anti-general GFP antibody was used to amplify the eYFP tag of the construct, Alexa 488) and colocalizing with vimentin, the tanycyte marker (Alexa 594). Tanycyte cell bodies and processes highly express the vector (indicated by arrowheads). The eYFP-marked cells have typical tanycyte morphology and coexpress vimentin. The anterior part was described as bregma −1.5 mm and posterior as bregma −1.85. 3V, third ventricle. (Scale bars, 50 and 20 μm [*Inset*].)

We observed that activation of single tanycytes could not only lead to a propagating wave of Ca^2+^ signaling in tanycytes but that activation of cells (*n* = 85) with small somata deeper in the parenchyma also occurred following this selective stimulation of a single tanycyte (*SI Appendix*, Fig. S1 and Movies S2 and S3). As these were labeled with Rhod-2 AM, we presumed them to be astrocytes, as these are preferentially labeled by Rhod-2 (28). We reasoned that if the activation of these parenchymal cells was a consequence of the activity in the stimulated tanycyte, then the occurrence of their activation should be temporally linked to that of the tanycyte. We therefore measured the time delay and normalized it to the distance between the stimulated tanycyte and the responding parenchymal cell (*SI Appendix*, Fig. S2). This showed that the activation of these presumed astrocytes occurred at a characteristic time (when normalized to distance from the tanycyte layer) that was consistent with direct activation by the stimulated tanycyte and indirect activation via a tanycyte that had been secondarily activated as part of the propagating wave of activity in the tanycyte layer (*SI Appendix*, Fig. S2). To understand how tanycytes activate parenchymal cells in more detail, we developed an optogenetic adenoviral construct, AdV-pTSHR-CatCh (Fig. 2), to permit their selective activation by blue light.

### Optogenetic Induction of Ca^2+^ Signals in Tanycytes.

To verify the specific expression of CatCh in tanycytes, we used immunohistochemical staining for the tanycytic marker vimentin to identify tanycytes. Following intracerebroventricular injections of AdV-pTSHR-CatCh, CatCh (tagged with enhanced yellow fluorescent protein; eYFP) was expressed in many tanycytes (Fig. 2 and *SI Appendix*, Fig. S3). This specificity of expression driven by the TSHR promoter has been described and characterized previously for a similar GCaMP3 construct (21). We never observed expression of CatCh in the pars tuberalis, showing that our viral transduction strategy was specific to the tanycytes (*SI Appendix*, Fig. S7) (21). Our viral transduction approach did not allow any discrimination of the tanycytic subtypes and thus all cells were equally transfected after viral injection. In this study, we focused on α_2_ and β_1_ cells as they are the closest partners to interact with NPY or POMC neurons in the ARC nucleus.

**Fig. 3.**
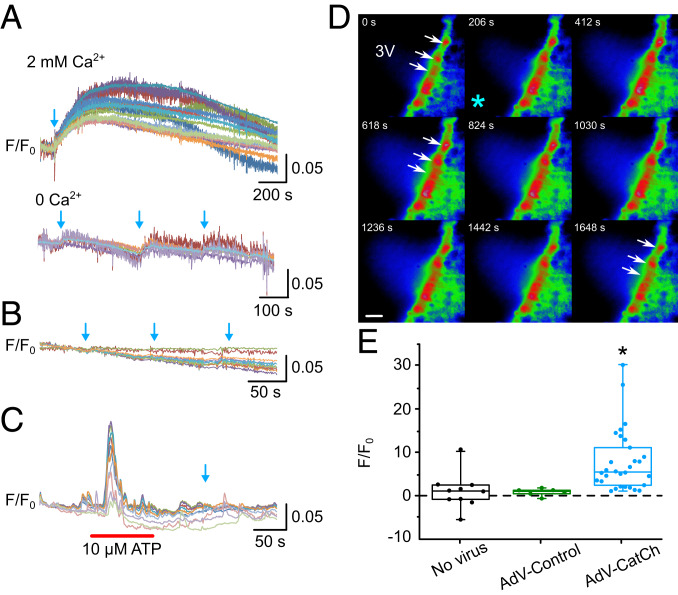
Optogenetic stimulation of tanycytes transduced with CatCh evokes long-lasting Ca^2+^ waves. (*A*) Intracellular Ca^2+^ (measured with Rhod-2) evoked by blue light (470-nm) optostimulation (indicated by the blue arrows). Each colored line represents an ROI, drawn around a single tanycyte cell body. In the second recording, removal of extracellular Ca^2+^ greatly attenuated the Ca^2+^ signal evoked by optostimulation. (*B*) In slices obtained from control animals injected with AdV-Control (pTSHR-GCaMP3, used as a control in the study), there was no effect of optostimulation on intracellular Ca^2+^. (*C*) To confirm the viability of tanycytes in the control brain slices, we used bath application of 10 μM ATP to trigger a Ca^2+^ response. Optostimulation did not produce any response (blue arrow). (*D*) Montage of pseudocolor images showing a response in tanycytes to the blue light optostimulation (delivered at the site of the asterisk). ROIs were drawn around individual tanycyte cell bodies to measure their activation. White arrows indicate tanycytes that respond to stimulation. (Scale bar, 50 μm.) Numbers in each image are the times in seconds after the beginning of recording. (*E*) Percent change in *F*/*F*_0_ evoked by optostimulation in sham controls (no virus), AdV-Control (GCaMP3 used as controls), and AdV-CatCh-transduced tanycytes. *P < 0.05. (The box represents the upper and lower quartiles, the central line the median, and the whiskers the maximum and minimum values.)

To confirm the functionality of the CatCh channel in tanycytes, we loaded mouse brain slices with the calcium indicator Rhod-2 AM. Tanycytes suitable for imaging were identified by the presence of the eYFP protein via fluorescence microscopy and by their characteristic morphology. Trains of blue light pulses (*Materials and Methods*; trains 10 to 20 s long) triggered long-lasting episodes of elevated intracellular Ca^2+^ in tanycytes (∼1,000 s with ≥1.05 units *F*/*F*_0_; Fig. 3 *A* and *D*). This elevation of intracellular Ca^2+^ depended upon Ca^2+^ influx through CatCh as, in the absence of added extracellular Ca^2+^, the blue light flashes caused only a very small and short-lived elevation of Ca^2+^ in tanycytes (Fig. 3*A*).

To ensure that the blue light itself did not trigger changes in intracellular Ca^2+^ in tanycytes, we examined slices from animals that had been injected with the control construct. In these slices, blue light did not evoke any Ca^2+^-mediated fluorescence signals in tanycytes (Fig. 3*B*). As tanycytes respond to the neurotransmitter ATP (19), we therefore applied 10 µM ATP in the bath as a positive control to demonstrate that tanycytes, which did not respond to blue light, were indeed capable of triggering Ca^2+^ signaling (Fig. 3*C*).

Taken together, these results demonstrate that selective expression of CatCh in tanycytes (AdV-CatCh) allows the specific activation of these cells via long-lasting Ca^2+^ signals that propagate through the tanycyte layer compared with control nontransduced tanycytes (no virus) or transduced with the control adenovirus vector that expressed GCaMP3 under the same promoter system (AdV-control; Fig. 3*E*; Kruskal–Wallis ANOVA, *P* = 0.000, followed by Dunn’s post hoc test control vs. CatCh, *P* = 0.003, no virus vs. CatCh, *P* = 0.001).

### Selective Activation of Tanycytes Induces Responses in Hypothalamic Neurons.

**Fig. 4.**
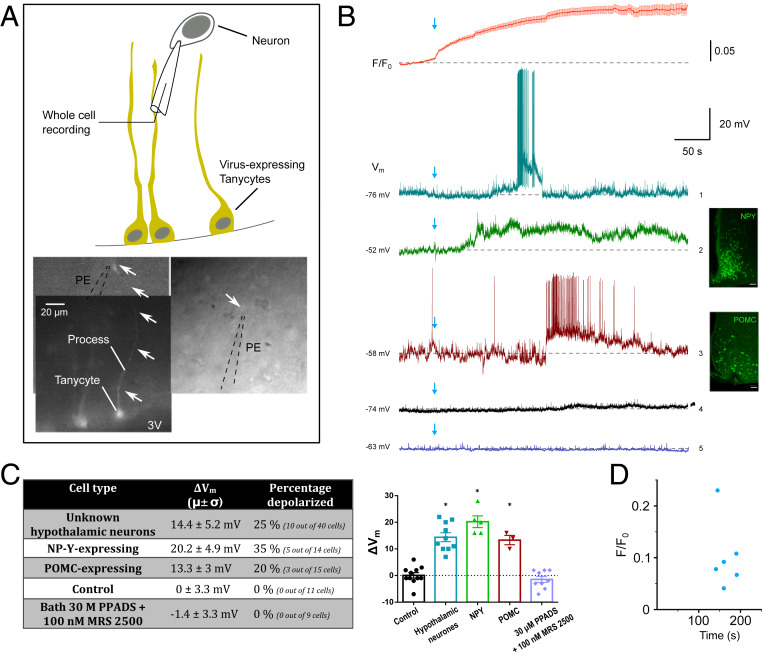
Optostimulation of tanycytes triggers depolarizing responses in arcuate nucleus neurons. (*A*) Schematic of the experimental design in acute brain slices expressing ectopic viral vectors (AdV-pTSHR-CatCh). The picture montage shows labeled tanycytes (eYFP fluorescence) with cell bodies and long processes. A recording was made from a neuron close to the end of the process (arrows; shown in bright-field images; PE, patch electrode). Recordings were made from neurons that were either close to the stained tanycytes (somata or processes) or close to the ependymal wall. Following establishment of recordings, the field of view was moved to ensure that the tanycyte cell bodies were exposed to blue light illumination. (*B*) Average (±SD) Rhod-2 Ca^2+^ imaging (red recording; *n* = 30) of tanycyte response to blue light optostimulation (indicated by the blue arrow; stimulation lasted for approximatively 15 s) in parallel with a whole-cell patch-current clamp recording from an arcuate neuron. Activation of tanycytes induced a current depolarization in close by hypothalamic neurons (turquoise recording 1; hypothalamic neurons). In some recordings, note the ramp depolarization evoked and burst of firing which was abruptly terminated. Similarly, in NPY-GFP–expressing animals, tanycyte activation induce a long current depolarization (green recording 2) as well as in POMC-GFP–expressing animals (red recording 3). (Scale bars, 50 μm.) Optostimulation had no effect on slices from control animals (black recording 4). In the presence of 100 nM MRS2500 (P2Y1 antagonist) and 30 μM PPADS (general P2 receptor blocker), optostimulation of tanycytes had no effect on the depolarization (purple recording 5). (*C*) The table summarizes the proportion of cells responding by a current depolarization after a tanycytic activation (average ± SD). Twenty-five percent of recorded neurons (10 out of 40 cells) responded. This induced on average a variation of the membrane potential for about 14.4 ± 5.2 mV. Similarly, 35% (5 out of 18 cells) of NPY- and 20% (3 out of 15 cells) POMC-expressing neurons showed a current depolarization, respectively, of 20.2 ± 4.9 and 13.3 ± 3 mV. We did not observe any change in membrane potential in control slices (from animals injected with a similar viral vector expressing GFP; 0.3 ± 3.4 mV). Bath application of a specific P2Y1 receptor antagonist (MRS2500) and a general P2 receptor blocker (PPADS) inhibited any membrane potential changes after tanycytic optostimulation (−1.4 ± 3.3 mV). The plot shows the variation between the baseline and at the peak of the depolarization of the membrane potential in each of the conditions (one-way ANOVA, *P* < 0.0001, followed by Tukey’s post hoc analysis, histogram shows mean ± SEM). (*D*) Plot of *F*/*F*_0_ vs. time of response in neurons (s). On average, neurons respond after 164.5 ± 21.5 s (average ± SD) with 0.1 ± 0.06 *F*/*F*_0_ (average ± SD).

To demonstrate communication between tanycytes and hypothalamic neurons, we used optogenetic stimulation of tanycytes while making visually guided whole-cell patch-clamp recordings from neurons in the arcuate nucleus (Fig. 4*A*). We examined whether there was an effect of tanycyte activation on the membrane potential of arcuate neurons. We predicted, from the temporal propagation of Ca^2+^ signaling along the tanycyte, that neurons located in the parenchyma at depths of 100 µm or more from the ventricle receiving input from tanycytes would take a minimum of 50 s to respond following stimulation of the tanycyte soma. To understand further the timing relation between the activation of the tanycytes and downstream activation of neurons, we recorded the changes in intracellular Ca^2+^ via Rhod-2 following optostimulation (Fig. 4*B*). As described earlier, we found that the Ca^2+^ signal recorded by Rhod-2 increased quite slowly following the blue light stimulation. Neuronal responses only occurred once the Rhod-2 signal in tanycytes had increased by 0.1 ± 0.06 *F*/*F*_0_ (mean ± SD of individual points corresponding to animals recorded; change in *F*/*F*_0_ vs. time of response in neurons is plotted in Fig. 4*D*).

Twenty-five percent of arcuate neurons (10 out of 40 cells recorded in 29 animals) showed a depolarization in their membrane potential following the optostimulation of tanycytes (Fig. 4*C*). This had a mean latency of 164.5 ± 21.5 s (*n* = 6 animals; mean ± SD) from the onset of blue light stimulation (Fig. 4*D*). The mean resting membrane potential of the recorded neurons was −59.6 ± 10.7 mV (mean ± SD; *n* = 10 cells in 10 animals). The mean depolarization observed was Δ*V*_m_ = 14.4 ± 5.2 mV (Fig. 4*B*, recording 1 and Fig. 4*C*; one-way ANOVA, *P* < 0.0001, followed by Tukey’s post hoc analysis). In slices from control-injected animals (GCaMP3 used as a control), optostimulation had little or no effect (Δ*V*_m_ = 0 ± 3.3 mV; average resting membrane potential, −53.7 ± 3.3 mV; mean ± SD; *n* = 10 cells in four animals; Fig. 4*B*, recording 4 and Fig. 4*C*). We conclude that tanycytes are able to communicate with nearby hypothalamic neurons and that optostimulation of tanycytes results in a sustained depolarization in about 25% of cells within the 300-µm-thick hypothalamic brain slice.

### Tanycytes Activate NPY and POMC Neurons.

There are two principal populations of neurons in the arcuate nucleus that regulate food intake—the NPY-containing orexigenic neurons, which are also GABAergic, and the POMC-containing anorexigenic neurons, which are mostly not GABAergic, but some are glutamatergic (29, 30). Understanding the extent to which tanycytes communicate with these neuronal populations is of critical importance in understanding how tanycytes might influence energy balance. We therefore used two transgenic mouse strains expressing green fluorescent protein (GFP) specifically in NPY-containing neurons and POMC-containing neurons to identify the neuronal phenotype for visually guided patch-clamp recording.

In acute slices prepared, we observed that 35% of NPY-GFP neurons recorded (5 out of 14 cells recorded in 10 animals) showed sustained depolarization in response to optogenetic stimulation of tanycytes (Fig. 4*B*, recording 2 and Fig. 4*C*). The average depolarization of membrane potential of NPY-GFP neurons was 20.2 ± 4.9 mV (mean ± SD; Fig. 4*C*; one-way ANOVA, *P* < 0.0001, followed by Tukey’s post hoc analysis, with a mean resting membrane potential of −60.6 ± 12.3 mV). Thus, tanycytes are able to excite NPY-containing neurons.

Similarly, in 20% of POMC-GFP neurons (3 out of 15 cells recorded in 11 animals; Fig. 4*B*, recording 3), the average depolarization of membrane potential observed was 13.3 ± 3 mV due to the optogenetic stimulation of tanycytes (mean ± SD; Fig. 4*C*; one-way ANOVA, *P* < 0.0001, followed by Tukey’s post hoc analysis, with mean resting membrane potential −56 ± 8 mV). Optostimulation of tanycytes ex vivo is able to activate both the orexigenic and anorexigenic pathways. We noticed that there was some heterogeneity in the responses evoked in neurons by tanycytes (e.g., presence of a depolarizing ramp and plateau) but these differences were not systematically related to neuronal phenotype (*SI Appendix*, Table S1).

### ATP Release from Tanycytes Is Required to Activate Arcuate Neuronal Networks.

To further dissect the mechanisms by which tanycytes might activate the arcuate networks, we tested whether this might be through the actions of ATP, known to be released by tanycytes both in the region of their cell bodies and deeper in the parenchyma (19, 22). We used a combination of a specific P2Y1 receptor antagonist (MRS2500) and a general P2 receptor blocker (pyridoxalphosphate-6-azophenyl-2',4'-disulfonic acid, PPADS; Fig. 4*B*, recording 5 and Fig. 4*C*). When bath applied, these antagonists prevented the ability of tanycyte activation to evoke depolarization in arcuate neurons (the average depolarization of membrane potential was −1.4 ± 3.3 mV; Fig. 4*C*; mean ± SD; *n* = 9 cells in eight animals). We further compared the proportions of neurons responding with depolarization to optogenetic stimulation of tanycytes in all CatCh-transduced, GCaMP3-transduced (used as a control) slices and in the presence of ATP receptor antagonists (Fig. 4*C*). Overall, these proportions were significantly different, giving confidence that tanycytes are indeed capable of activating neurons via an ATP receptor-dependent mechanism (χ^2^ = 6.54, degrees of freedom 2, *P* = 0.038).

### Tanycyte Activation Induces Hyperphagia in Animals.

**Fig. 5.**
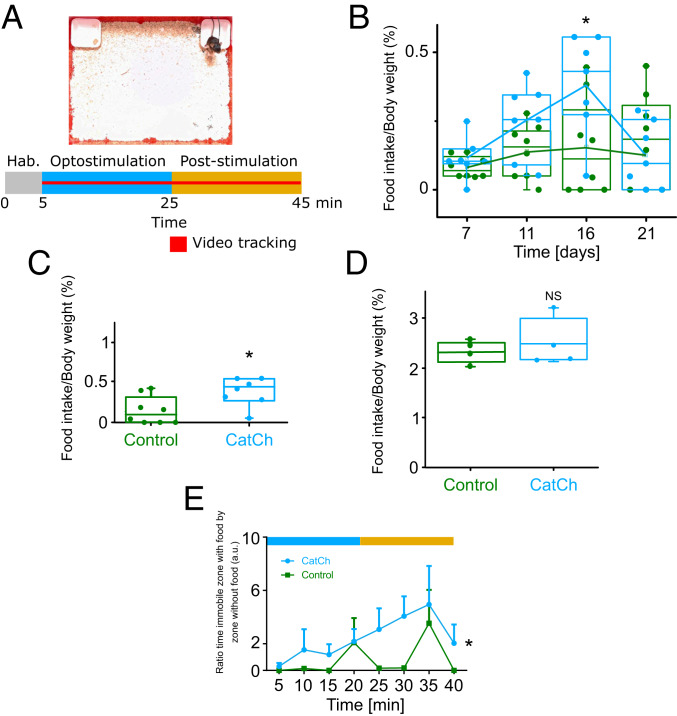
Optostimulation of tanycytes triggers an acute hyperphagia. (*A*) Optostimulation of tanycytes had an effect on food intake during the 40-min test. Animals were in an open field with two cups (changed alternately) that contained a slightly enriched pellet. Stimulation occurred for 20 min at the same frequency as the in vitro study and for 20 min left free-running. Video tracking was used to analyze animal behavior. (*B*) Time course of the behavior response after activation of tanycytes by CatCh versus controls. Adenovirus vector expression seems to be cleared by cells after 21 d postinfection. The optimal period for the expression is around 16 d (two-way repeated-measures ANOVA, *P = 0.004, followed by Sidak’s post hoc analysis; *n* = 8 and 7 mice per group; for all box plots, the box represents the upper and lower quartiles, the central line the median, and the whiskers the maximum and minimum values; the open square indicates the mean). (*C*) The optostimulation of tanycytes induces an acute hyperphagia in animals when received between ZT 7 and 10 (Mann–Whitney *U* test, *P = 0.02; *n* = 7 mice in each group, the box represents the upper and lower quartiles, the central line the median, and the whiskers the maximum and minimum values). (*D*) Nineteen hours of fasting prior to the test abolished the effect of tanycyte activation on food intake (ZT 7 to 10; Mann–Whitney *U* test, *P* > 0.05; *n* = 4 mice per group, the box represents the upper and lower quartiles, the central line the median, and the whiskers the maximum and minimum values). No effect was observed if animals were acutely fasted 1 h prior to the test (*SI Appendix*, Fig. S5*A*). NS, not significant. (*E*) The ratio of the time spent immobile in the food zone to the time spent immobile in the nonfood zone during the test. Activation of tanycytes will trigger an acute hyperphagia (time spent immobile “eating”) after approximatively 25 min (Friedman test, *P = 0.02; *n* = 7, mean ± SEM).

The capacity of tanycytes to activate both opposing pathways of the hypothalamic networks is puzzling. We therefore investigated next how tanycytes altered feeding behavior to determine the net effect of their activation in vivo. Animals were placed in an open field-type chamber and presented with food slightly enhanced in palatability (increased by 5.5% in fatty acids and 7% in protein standard chow) as shown in Fig. 5*A*. Video tracking of the animal was performed for 40 min (after a short habituation period) and optostimulation was performed for the first 20 min of this 40-min session. The amount of food eaten during that entire episode was measured and normalized to the animal’s body weight. Tanycyte stimulation under this paradigm induced an acute increase in food intake during the test (Fig. 5 *B* and *C*) but not in the long term (*SI Appendix*, Fig. S6).

As we used adenovirus which has a transitory expression and should be cleared by the cells after about 3 wk, we first studied the time course of the phenotypic effects of AdV-CatCh expression. We therefore optostimulated virally transduced mice (CatCh and controls) every day for 21 d between zeitgeber time (ZT) 7 and 10. We found that the phenotype took 11 d to develop, reached its peak at 16 d postinjection, and then dissipated, as the virus was cleared after 21 d (Fig. 5*B*; two-way ANOVA, *P* < 0.004, followed by Sidak’s post hoc analysis). Immunohistology for GFP indeed demonstrated the absence of any viral expression after 4 wk (*SI Appendix*, Fig. S3). To eliminate the possibility that the enhanced food intake occurred after the optostimulation ceased (a rebound effect from hypothesized inhibition of feeding during the optostimulation), we made recordings for 40 min where food was available for the entire 40 min but the optostimulation occurred only in the last 20 min of the session. Food intake was still increased (*SI Appendix*, Fig. S4), suggesting that activation of tanycytes does indeed increase food intake.

We then used animals between 11 and 16 d postsurgery to demonstrate that the response in animals was greater when animals received the optostimulation between ZT 7 and 10 (Fig. 5*C*; Mann–Whitney *U* test, *P* = 0.02; for *n* = 7 mice per group). Furthermore, after an overnight 19-h fasting period, optostimulation at the same period of the day had no effect on food intake (Fig. 5*D*; Mann–Whitney *U* test, *P* = 0.11; for *n* = 4 mice per group).

To gain further evidence of the behavioral effects of tanycyte activation, we not only quantified the food intake but also the time spent immobile in the food zone versus that spent in the nonfood zone—considered here as a proxy measure of time spent feeding. Optostimulation of tanycytes caused the CatCh mice to spend more time in the food zone compared with the control mice. This effect reached a peak after ∼25 min from the beginning of the optostimulation (Fig. 5*E*; Friedman test, *P* = 0.02). This corresponds to the beginning of the poststimulation period of 20 min.

## Discussion

Tanycytes have a unique morphology which enables them to contact simultaneously the CSF and major neuronal populations in the hypothalamic parenchyma. A growing body of evidence is highly suggestive that tanycytes could have a fundamental role in regulating the neuronal networks controlling appetite. Prior work has shown that tanycytes sense nutrients such as glucose, amino acids, and fatty acids (19–23, 27). Information about the nutritive status of an individual is important for energy balance. Nevertheless, demonstration that tanycytes could directly modulate neurons and thus pass on this metabolic information has been lacking. We have now shown that tanycytes can activate hypothalamic arcuate neurons.

Tanycytes are known to make close anatomical connections to NPY neurons (31). Close apposition to the arcuate POMC neurons has not yet been described. Our data showing that tanycytes can induce changes in membrane potential of NPY neurons are consistent with this previous study. However, we also demonstrated that tanycytes can induce membrane potential changes in POMC-expressing neurons. Recently, the distinction between AgRP/NPY and POMC neurons in the arcuate has been blurred as some neurons identified by POMC promoter-driven GFP expression also express AgRP (27% of the total) (32). It is therefore possible that POMC+ neurons that are activated by tanycyte stimulation could be exclusively from this population. However, at this stage, the more probable hypothesis (simply on grounds of frequency of the POMC+ subtypes) is that tanycytes activate both the orexigenic and anorexigenic pathways. The tanycyte-to-neuron communication we document here is rather slow and relatively long-lasting. These aspects are most similar to volume transmission mediated via ATP release from tanycytes that diffuses in the extracellular space and activates P2 receptors known to be expressed on arcuate neurons and to influence food intake (33–40).

### Ca^2+^ Signaling in Tanycytes.

We exploited the brief infrared stimulation of ROIs to explore the signaling capacity of individual tanycytes. This showed that activation of just a single tanycyte could evoke Ca^2+^ mobilization in the neighboring tanycytes and a spreading wave that on average traveled a distance of a few tanycytes in either direction (dorsal or ventral) but could on occasion spread as far as eight tanycytes. This wave spread at a slow pace of just over 1 µm/s. In addition, stimulation of the tanycyte soma caused a traveling Ca^2+^ wave along the tanycyte process of around 2.4 µm/s. These observations have two important consequences for optogenetic stimulation of tanycytes. First, even if only a few tanycytes in a slice express CatCh, we would expect their stimulation to evoke activation of the neighboring tanycytes. This is likely to enhance the efficacy of the optogenetic stimulation. Second, as Ca^2+^ signals evoked in the soma propagate along the process, optogenetic stimulation of CatCh in the soma will plausibly be able to activate the neuronal networks deeper in the soma. This raises the possibility that small domains of tanycytes (anteroposterior and ventrodorsal irrespective of their subtypes) could be preferentially linked to populations of neurons in the parenchyma (expressing a particular purinergic receptor) and activating a neuronal pathway (motivated feeding, metabolic cues, foraging, etc.) (41).

Blue light-induced activation of CatCh expressed in tanycytes evoked a slowly rising and long-lasting Ca^2+^ signal in the tanycytes. This Ca^2+^ signal depended upon an influx of Ca^2+^ presumably through the CatCh channels themselves. We found that once the Rhod-2 AM signal showed that intracellular Ca^2+^ had increased by at least 10% above the prestimulus baseline, the tanycytes were able to evoke responses in neurons. We thus used a wide time window of up to 350 s, based on the time it took for the Ca^2+^ signal to exceed this threshold and the time the signal was maintained above this threshold, to analyze the effect of tanycytes on neuronal activity.

### Tanycytes Activate Arcuate Neurons.

We found that optogenetic stimulation of tanycytes could induce a substantial depolarization in hypothalamic neurons that was sufficient to trigger firing. On occasion, tanycytes appeared to induce plateau-like potentials in arcuate neurons—a depolarization that would end in abrupt repolarization (Fig. 4*B*). Our findings further suggest that tanycytes can activate multiple neuronal types in the arcuate including, but not limited to, NPY+ and POMC+ subtypes. It is possible that the activation of the arcuate neurons by tanycytes was indirect via an interposed neuronal population. Given the restriction of the tanycyte processes to the arcuate, ventromedial nucleus and median eminence, such a population would have to be localized to the vicinity of the third ventricle. Nevertheless, the possibility of an unknown interposed neuronal population does not weaken our conclusion that activation of tanycytes leads to the activation of arcuate neurons including those of the NPY and POMC subtypes.

As tanycytes are capable of releasing ATP in response to activation by glucose or amino acids (19, 22), ATP is thus a candidate transmitter for mediating their actions on hypothalamic neurons. ATP receptors are widely expressed on hypothalamic neurons of the arcuate nucleus and ventromedial nucleus, and have been linked to regulation of feeding, making this a plausible hypothesis (35, 38, 39). P2X4 receptors are expressed by the NPY/AgRP neuron receptors; their activation increases spontaneous GABA release from these cells onto the POMC-containing neurons (39). We never observed an effect of tanycyte activation on the membrane potential of arcuate neurons in the presence of P2 receptor antagonists. Thus, ATP release from tanycytes may mediate an excitatory action to both the NPY/AgRP and POMC neurons of the arcuate. This action of tanycytes on POMC neurons, even although it is potentially ATP receptor-mediated, is distinct from the P2X4-mediated action of exogenously applied ATP which instead increases GABAergic inputs to POMC neurons (39). We cannot exclude that additional signaling agents could be released from tanycytes that could further contribute to neuronal activation.

### Effects of Glial Cells on Arcuate Networks.

Two previous studies have expressed designer receptors exclusively activated by designer drugs (DREADD) constructs in GFAP+ cells of the arcuate (9, 10). These constructs were mainly expressed in astrocytes; however, this targeting strategy also transduced some tanycytes (10). The findings from these two groups differ. Yang et al. found that activation of the transduced astrocytes (via clozapine-*N*-oxide [CNO], the agonist for DREADD) reduced food intake via an adenosine A_1_ receptor-mediated inhibition of the NPY/AgRP neurons (9). However, Chen et al. found that CNO injection caused depolarization of the NPY neurons and increased feeding (10). Chen et al. reported that activation of the GFAP+ cells had no net effect on POMC neurons (10). Thus, activation of (mainly) astrocytes had an orexigenic effect via unopposed activation of the NPY neuronal pathway.

Our results differ in some respects from both of these studies but, like Chen et al., we found that activation of tanycytes increased feeding. In our study, we specifically targeted our constructs to the tanycytes. Tanycytes activate both NPY and POMC neurons with roughly equal efficacy. Although we found that tanycytes could indeed activate astrocytes (*SI Appendix*, Figs. S1 and S2), our observations cannot simply be explained as an indirect effect of tanycytes acting via astrocytes. Whereas this pathway might contribute to the observed activation of the NPY neurons (10), the activation of POMC neurons is most likely mainly tanycytic.

### Tanycytes Contribute to the Control of Appetite but Are Unlikely to Change the Homeostatic Balance When Acutely Activated.

The arcuate nucleus contains two major opposing neuronal pathways involved in the control of appetite and energy balance. One of these pathways is the orexigenic pathway involving the NPY/AgRP-containing neurons which increase food intake and energy storage by increasing adiposity. The other pathway is anorexigenic and involves the POMC-containing neurons and a recently discovered population of non–POMC-containing glutamatergic neurons (30). Activation of these anorexigenic neurons decreases appetite, increases energy expenditure, and thus reduces adiposity. As tanycytes respond to elevated glucose (19–21), increases in amino acid concentration (22), and levels of fatty acids (23), which would be elevated in plasma and CSF following a meal, it would be physiologically adaptive if tanycytes were to act to reduce appetite by activating the anorexigenic pathways.

It is thus surprising that we found that tanycytes induce an acute hyperphagia in an ad libitum-fed state. However, this is consistent with the observation of the activation of the NPY-expressing population. Abundant evidence suggests that activation of the NPY/AgRP neurons results in an immediate increase in feeding (7, 42). By contrast, actions of the hypothalamic POMC network in countering appetite are slow (43). Thus, timing of activity within the neural network could be key to understanding how tanycyte activation regulates food intake. However, if tanycytes also activated the recently discovered glutamatergic population in the arcuate, this would rapidly counteract the activation of the NPY/AgRP neurons. Furthermore, no investigations have yet been done on the role of “hybrid” AgRP/POMC-expressing hypothalamic neurons discovered recently (32).

Interestingly, long fasting (Fig. 4*D*) as well as acute fasting (1 h prior to activation; *SI Appendix*, Fig. S5) prevent the effect of optostimulation of tanycytes on feeding. This could be partially explained by the effect of sensory cues on orexigenic neurons described previously (4–6, 44). Indeed, sensory cues informing the animals of food availability will induce a rapid inhibition of the orexigenic neurons. The orexigenic pathway is thus highly activated prior to food intake and inactivated during consummatory episodes. Following a period of fasting, when the orexigenic drive is strong, activation of tanycytes may not add further to this and their contribution could thus be masked—a ceiling effect. No effect on long-term body weight or food intake was observed (monitored days after in vivo activation; *SI Appendix*, Fig. S6), indicating that this brief (20-min) stimulation of tanycytes does not affect general energy homeostasis.

In addition to measuring the amount of food consumed during the test, we measured the ratio of time immobile in the food zone versus the nonfood zone. This is a continual measure that can be considered as an approximate proxy measure of food intake and appetite (Fig. 4*E*). This analysis of the time spent in the food zones showed that it required relatively long activation (20 min) of tanycytes to change this ratio in favor of spending time immobile in the food zone. This in turn suggests that the effects of tanycytes on appetite are slow to induce hyperphagia.

### Optostimulation versus Physiological Stimulation of Tanycytes.

The optostimulation of tanycytes is a strong and nonphysiological stimulus. As such, it indicates what tanycytes may be capable of contributing to the physiological control of energy balance. However, physiological stimuli such as glucose or amino acids could trigger different actions. Although glucose triggers tanycyte responses, these occur only via P2Y1 receptors (19, 21) and release of ATP via Cx43 (20). By contrast, amino acids evoke tanycyte responses that depend on ATP but require the P2Y1 receptor and additional P2 receptors (22). Indeed, the mechanism of ATP release for amino acids depends on the activating amino acid (pannexin 1 for arginine and CalHM1 for alanine) (22) and differs from that which underlies glucose-induced responses (20). Tanycytes thus have subtly different ATP-dependent signaling pathways depending upon the activating stimulus. It is probable that optogenetic stimulation, by causing a powerful and nonlocalized Ca^2+^ influx, will activate all of these pathways somewhat indiscriminately. Physiological stimuli, by selectively activating these different pathways, could potentially have more selective effects on feeding and energy homeostasis compared with the optogenetic stimulation. Nevertheless, our data demonstrate that tanycytes are capable of activating arcuate neurons and altering feeding behavior when activated via an optogenetic stimulus.

Our experiments highlight the role of tanycytes in vivo in rodents. Tanycytes are present in humans (45–47) but little is known about their role in food intake and nutrient sensing. These important cells may thus represent a new target for developing mechanistically informed strategies to assist in the maintenance of healthy body weight in the human population.

## Materials and Methods

### Experimental Model and Characteristics.

All experiments and procedures in this study were performed in strict accordance with the UK Animals (1986) Scientific Procedures Act and the project was approved by the Animal Welfare and Ethical Review Board of the University of Warwick and the UK Home Office.

Animals were housed in same-sex sibling groups in controlled temperature and light–dark cycles (12:12 h) with ad libitum food and water. For fasting experiments, food pellets were removed from the animal’s cage from 1700 to 1200 the next day or in short-term fasting, 1 h prior to behavioral tests. Males and females, wild-type C57BL/6, NPY-GFP B6.FVB-Tg(Npy-hrGFP)1Lowl/J, and POMC-GFP C57BL/6J-Tg(Pomc-EGFP)1Lowl/J mice (both strains obtained from the Jackson Laboratory) were aged between 9 and 25 wk.

### Intracerebroventricular Injections and Optic Fiber Implantation.

Animals were injected in the third ventricle between 9 and 12 wk. The procedure was done in strict accordance with the UK Animals (1986) Scientific Procedures Act. Animals were maintained under deep anesthesia via inhalation of isoflurane (Baxter). The level of anesthesia was verified by testing of paw and tail withdrawal reflexes regularly during the procedure. The animals were placed in a stereotaxic frame (Kopf). A small hole was drilled in the skull to permit injection (via a 5-μL calibrated microcapillary tube; Sigma) of viral vectors into the third ventricle (2.5 to 5 × 10^9^ viral particles) at stereotaxic coordinates: bregma −1.79 mm; midline 0 mm; dorsal surface −5.85 mm. During the procedure, a single injection of Metacam (meloxicam) injectable (5 mg/mL; Boehringer Ingelheim) was given subcutaneously to the animal. The animals recovered for a week, and then acute slices were made.

Fiber implantation after viral infection was done in the lateral ventricle (stereotaxic coordinates: bregma 0 mm; midline 0.75 mm; dorsal surface 2.3 mm). The same transfection was obtained with the same quantity injected. A second small hole was drilled posteriorly (stereotaxic coordinates: bregma −1.79 mm; midline 0 mm; dorsal surface −5.85 mm) and an optic fiber was implanted (Thorlabs; CFML12L05 cannula 200 μm) and securely sealed with dental cement to the skull. During surgeries the experimenter was blind to the viral type that was being injected and this was only revealed at the end for statistical analysis to avoid any bias.

### Acute Slice Preparation.

Animals were humanely killed by cervical dislocation in accordance with schedule 1 of the Animals (Scientific Procedures) Act 1986. The brain was rapidly dissected and placed in ice-cold artificial cerebrospinal fluid (aCSF; 124 mM NaCl, 26 mM NaHCO_3_, 1.25 mM NaH_2_PO_4_, 3 mM KCl, 2 mM CaCl_2_, 1 mM MgSO_4_, 10 mM glucose, saturated with 95% O_2_/5% CO_2_) with an additional 10 mM MgCl_2_. Coronal sections 300 µm thick were prepared using a vibrating microtome (Microm; HM650). Each section was subsequently dissected along the midline separating the third ventricle and incubated in 36 °C aCSF for 60 min to allow for recovery of adenine nucleotide levels (48). Slices were then transferred to 1 or 10 mM glucose aCSF (osmolarity maintained by the addition of 9 mM sucrose) at room temperature for storage until required.

### Ca^2+^ Imaging and Optogenetic Stimulation.

Hypothalamic slices were incubated with the Ca^2+^ indicator Rhod-2 AM (12.5 µg/mL in 0.125% dimethyl sulfoxide, 0.025% pluronic) for 30 min in either 1 or 10 mM glucose in aCSF. Loaded slices were mounted onto a Scientifica Slicescope and observed via an Olympus 60× water-immersion objective (numerical aperture [NA] 1.0). Illumination was provided via a 470-nm light-emitting diode (LED) (OptoLED; Cairn Research) and a Hamamatsu ImageEM electron multiplying charge-coupled device camera was used to collect the images. MetaFluor imaging software was used to control the illumination and camera in all experiments. Optogenetic stimulation via blue light (470-nm LED) was performed as a series of 20-ms flashes separated by 50-ms intervals over approximatively 15 s (49). Recordings were performed at 28 to 30 °C, and the recording chamber was perfused at 3 mL/min.

For the in vivo optostimulation, the same pattern of intervals for the flashes was used. A FiberOptoMeter (FOM-02DM; Npi; 470-nm LED) linked to a Master-8 (A.M.P.I.) permitted the stimulation in vivo.

### Multiphoton Imaging and Experiments.

Slices were loaded with Rhod-2 AM as previously described and transferred to the multiphoton imaging rig. Multiphoton imaging and stimulation experiments were performed using a 7MP Zeiss multiphoton microscope using a 20× objective (NA 1.0) coupled with a Mai Tai Spectra-Physics pulsed femtosecond near-IR (infrared) eHP imaging laser. Zen 2009 imaging software was used to control the experiments.

To investigate Ca^2+^ wave spread between tanycytes and along processes, an ROI was drawn around the excited cell and this was then scanned with a second IR laser at a wavelength of 720 to 730 nm for a few seconds to cause Ca^2+^ release within the selected ROI. For analysis, ROIs were placed around all neighboring tanycytes that responded to initial excitation and at intervals along observable processes. The distance from the excited cell (center of the cell) to the nearest edge of the most distant responding tanycyte ROI (both dorsal and ventral) or the furthest ROI along the process and the time between initial excitation to induction of response in the most distant responding cell or furthest distance along the process was measured to calculate the speed of signal propagation.

### Electrophysiological Recordings.

Hypothalamic neurons, anatomically close to nearby tanycytes expressing the viral construct, were recorded in the whole-cell patch configuration as depicted in Fig. 4*A*. An Axopatch 200B amplifier was used to record cells in the current-clamp mode. The data were acquired at 3.3 kHz per channel and filtered at 1 kHz. The cells were recorded in aCSF (124 mM NaCl, 26 mM NaHCO_3_, 1.25 mM NaH_2_PO_4_, 3 mM KCl, 2 mM CaCl_2_, 1 mM MgSO_4_, 10 mM glucose, saturated with 95% O_2_/5% CO_2_). The patch electrodes were filled with a solution that comprised 130 mM K-gluconate, 10 mM KCl, 2 mM CaCl_2_, 10 mM EGTA, 10 mM Hepes, adjusted to pH 7.3 with KOH, and a final osmolarity of 295 mOsm. Cells were excluded from analysis if the recordings exhibited an obviously unstable resting potential. Most of the recordings were performed around bregma −1.7/−1.9 mm.

### Immunohistochemistry and Adenoviral Vector Construction.

After recording, slices were fixed overnight (about 16 h) in 4% formaldehyde in 0.1 M phosphate buffer and then washed with a phosphate-buffered saline (PBS) solution three times for 15 min each. Slices were blocked in PBS, 5% bovine serum albumin, 0.4% Triton X-100 for 1 h at room temperature. Primary antibodies were incubated for 2 h at room temperature: vimentin (tanycyte marker) 1:500 (Abcam; ab24525) and GFP (for the viral construct) 1:500 (Abcam; ab6556); the same constructs were already characterized for cellular specificity in ref. 21. Slices were then washed three times in PBS and incubated with a fluorescent conjugated secondary antibody (goat anti-rabbit or goat anti-mouse; 1:1,000; Invitrogen). Three final washes in PBS were done and slices were mounted in VectaShield with DAPI (Vector Labs) on a microscope slide. Imaging was performed on a Leica SP5 confocal microscope.

Adv-pTSHR-CatCh and the control (GFP) were made by previously established methods (50) that involved cloning the 5′ flanking region of the rat TSH receptor gene (51) and mutagenesis of the channelrhodopsin gene (52) into a dual-promoter construct (53).

### Behavioral Studies.

Animals were habituated to an open field-type test for a full week prior to trials (red cage of 480 × 375 × 210 mm). Tests were done approximately in the middle of the light phase (between ZT 7 and 10). Animals were tested every week until viral expression was not observed.

Two zones were delimited in the cage on opposite corners (Fig. 4*A*; small white plastic cups). A slightly more palatable food (normal chow increased by 5.5% in fatty acids and 7% in proteins) was presented in the zone (randomly alternated). Animals were attached to the optic cable via the cannula of the implanted optic fiber on the skull. The test started 5 min after the animal was introduced into the field. Optostimulation by blue light was induced for 20 min following the pattern used for the ex vivo study (470-nm LED, 20-ms flashes separated by 50-ms intervals). Video tracking of the animal was continuously done for 40 min.

After the trial, animals were unplugged and the amount of food eaten during the entire episode was recorded. The food weight was normalized to the animal’s body weight.

Videos of animals’ behaviors were analyzed by the tracking software ANY-maze. For the immobile episodes in the food versus nonfood zone, we used the software features “calculated by summing the duration of each visit to the zone where a visit starts at the time of a zone entry and ends at the time of a zone exit.” The phenotypes of the animals were only revealed to the experimenter at the end of the analysis.

### Quantification and Statistical Analysis.

Data are shown using boxplots (median and interquartile range with whiskers representing the outlier at 1.5× interquartile range). When data fail to meet normality and homoscedasticity criteria, nonparametric statistics were used.

Nonparametric Mann–Whitney *U* test was used to test data when two distributions were compared. For more than two, we used the Friedman test. For parametric analysis, we used one- and two-way ANOVA followed by either Sidak’s or Dunnett’s post hoc tests for intergroup comparisons. χ^2^ was used to compare depolarization of neuron type.

Stimulation of an individual tanycyte or group of tanycytes, monitored via whole-cell recording, was deemed to be an independent replicate. In practice, in almost all cases only one neuron was recorded per slice.

### Materials and Data Availability.

The data that support the findings of this study are available within *SI Appendix*. Reagents are available from the corresponding author upon reasonable request.

## Supplementary Material

Supplementary File

Supplementary File

Supplementary File

Supplementary File

Supplementary File
