# Supplementary material for: Hypothalamic tanycytes generate acute hyperphagia through activation of the arcuate neuronal network
Source: Proc Natl Acad Sci U S A. 2020 Jun 8;117(25):14473–81. doi: 10.1073/pnas.1919887117 (PMC7322081; doi:10.1073/pnas.1919887117)
Supplement: Supplementary File [file pnas.1919887117.sapp.pdf]

Supplementary Information for

## Hypothalamic tanycytes generate acute hyperphagia through activation of the arcuate neuronal network.

Matei Bolborea*, Eric Pollatzek, Heather Benford, Tamara Sotelo-Hitschfeld and Nicholas Dale*

* Co-corresponding authors: Dr. Matei Bolborea and Prof. Nicholas Dale
Email: m.bolborea@warwick.ac.uk
Email: n.e.dale@warwick.ac.uk

**This PDF file includes:**

Figures S1 to S7
Tables S1
Legends for Movies S1 to S3

**Other supplementary materials for this manuscript include the following:**

Movies S1 to S3

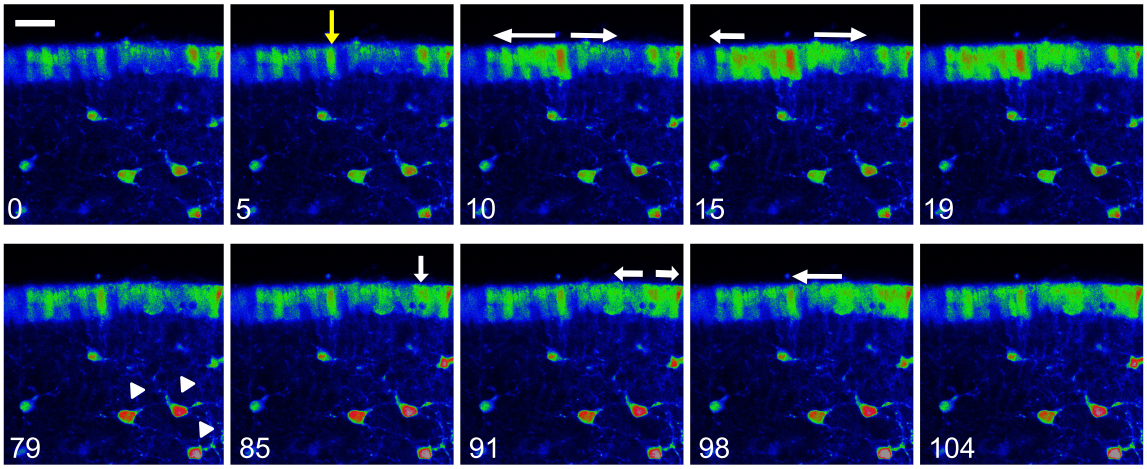


**Fig. S1. Propagation of $Ca^{2+}$ wave from a single tanycyte to multiple neighboring tanycytes and cells deeper in the parenchyma**:

Montage of images of Rhod-2 loaded cells, showing the tanycyte layer and small cells deeper in the parenchyma. The tanycyte indicated by yellow arrow was stimulated by IR light and this evoked a spreading wave of $Ca^{2+}$ activity in neighboring tanycytes (white horizontal arrows). Cells deeper in the parenchyma (almost certainly astrocytes, white arrow heads) showed an elevation of $Ca^{2+}$ some 70 s after the IR stimulation of the single tanycyte. At 85 s a secondary wave of activity spontaneously initiated (white vertical arrow) which then spread along the layer (horizontal white arrows). Supplementary Movie 2 shows this response. Slices from wild-type mice (not injected with CatCh) were used for these experiments. Scale bar 20 µm.

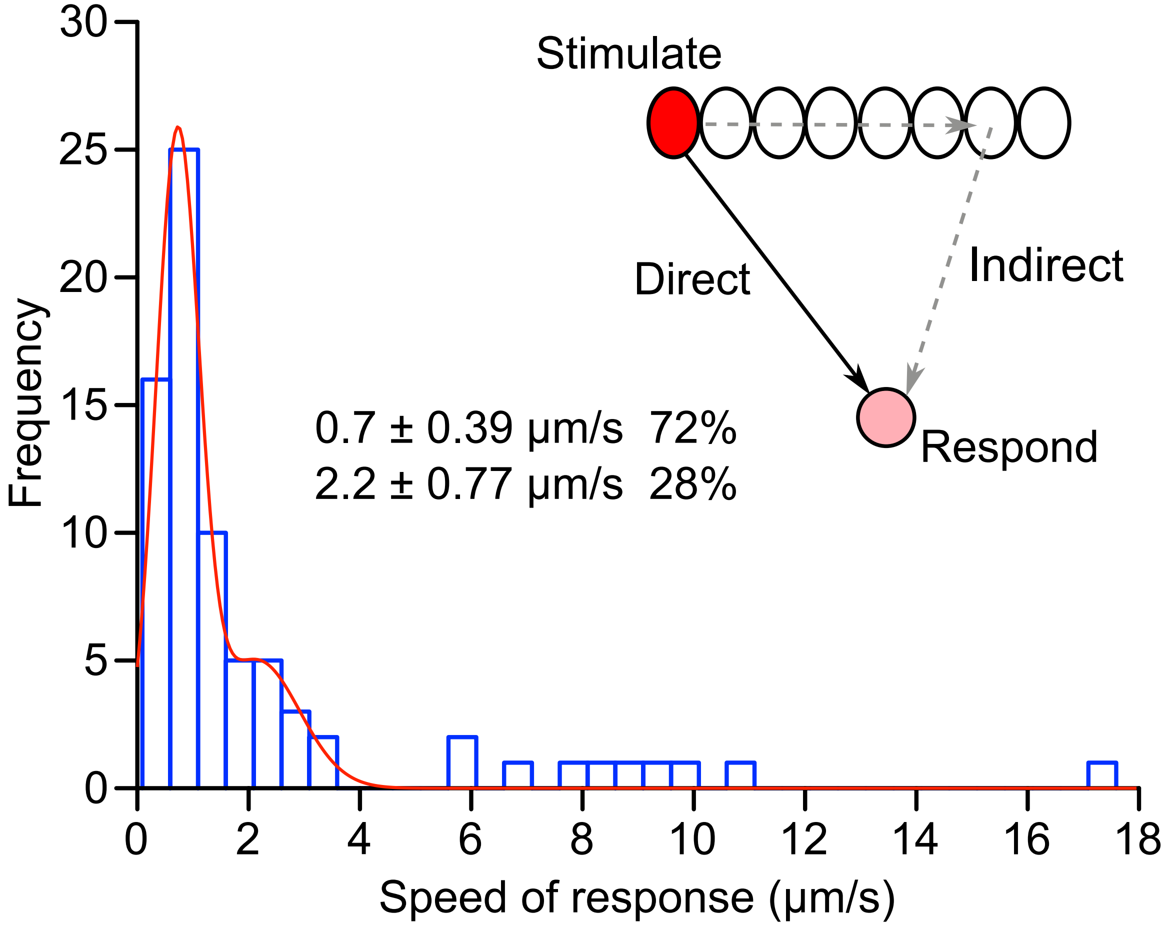


**Fig. S2. Activation of parenchymal cells by IR stimulation of a single tanycyte shows timing characteristic of direct and indirect activation**:

Histogram showing the speed of activation (distance between stimulated tanycyte and activated parenchymal cell divided by the time delay between stimulation of the tanycyte and the activation of the parenchymal cell) plotted for 76 cells (most likely astrocytes) that showed increased $Ca^{2+}$ signalling following the IR stimulation of the tanycyte. The histogram could be fitted with two gaussian distributions (red line) one with a mean speed of 0.7 µm/s and a second with a mean speed of 2.2 µm/s. The faster speed of activation (28% of interactions, by area under the gaussian) is consistent with a direct pathway of activation (inset cartoon) taking into account the speed of propagation of the $Ca^{2+}$ wave along the tanycyte process. The slower speed of activation is consistent with indirect activation from a tanycyte secondarily activated by the IR stimulation (cartoon).

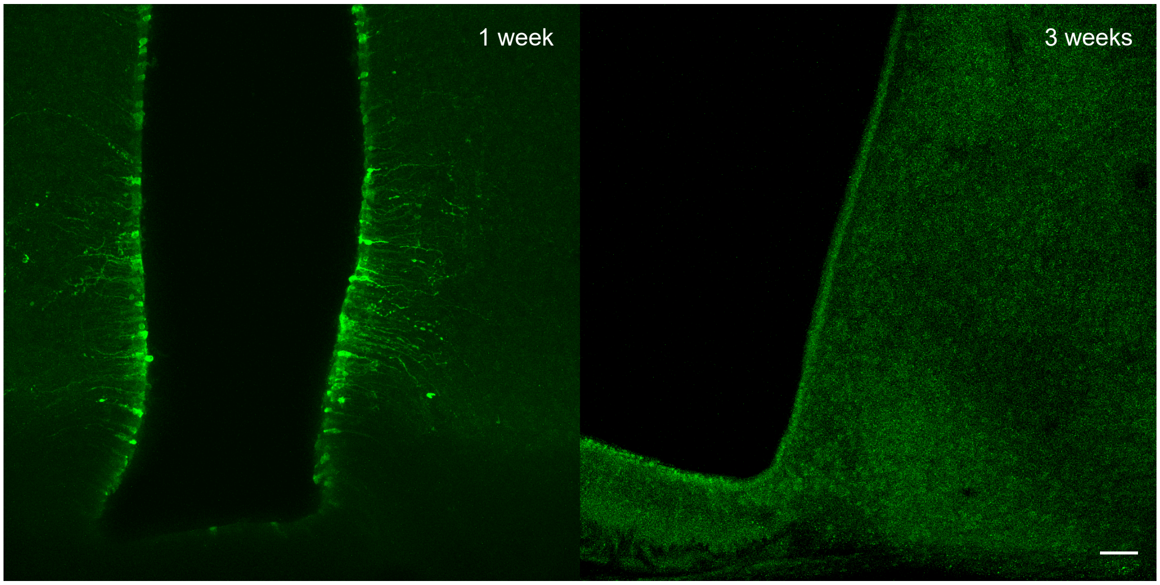


**Fig. S3. Time course of adenoviral expression**:

Immunohistochemistry of hypothalamic sections expressing viral vector (AdV-pTSHR-CatCh; an anti-general GFP antibody was used to amplify the eYFP tag of the construct, Alexa 488) after 1 week and 3 weeks incubation, 2 weeks is shown in Figure 1. Lost and/or decreased vector expression was observed after 3 weeks. Scale bar represents 50 μm.

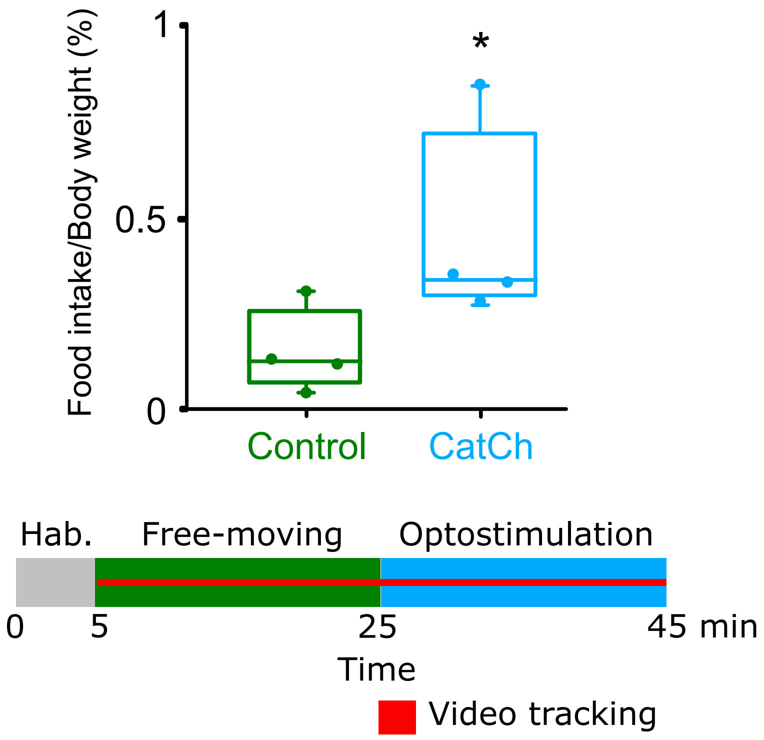


**Fig. S4. Delaying optostimulation to second half of 40 minutes recording and availability of feed still increases food intake**:

After the habituation period animals received the food pellet. Optostimulation was performed as previously, but after a delay of 20 minutes. The trial was stopped after a total of 45 minutes. Food intake was recorded at the end of the period. The tanycyte optostimulation had a significant effect on food intake even although it was delayed by 20 minutes with respect to the availability of food (Mann-Whitney test $p_{value} = 0.02$).

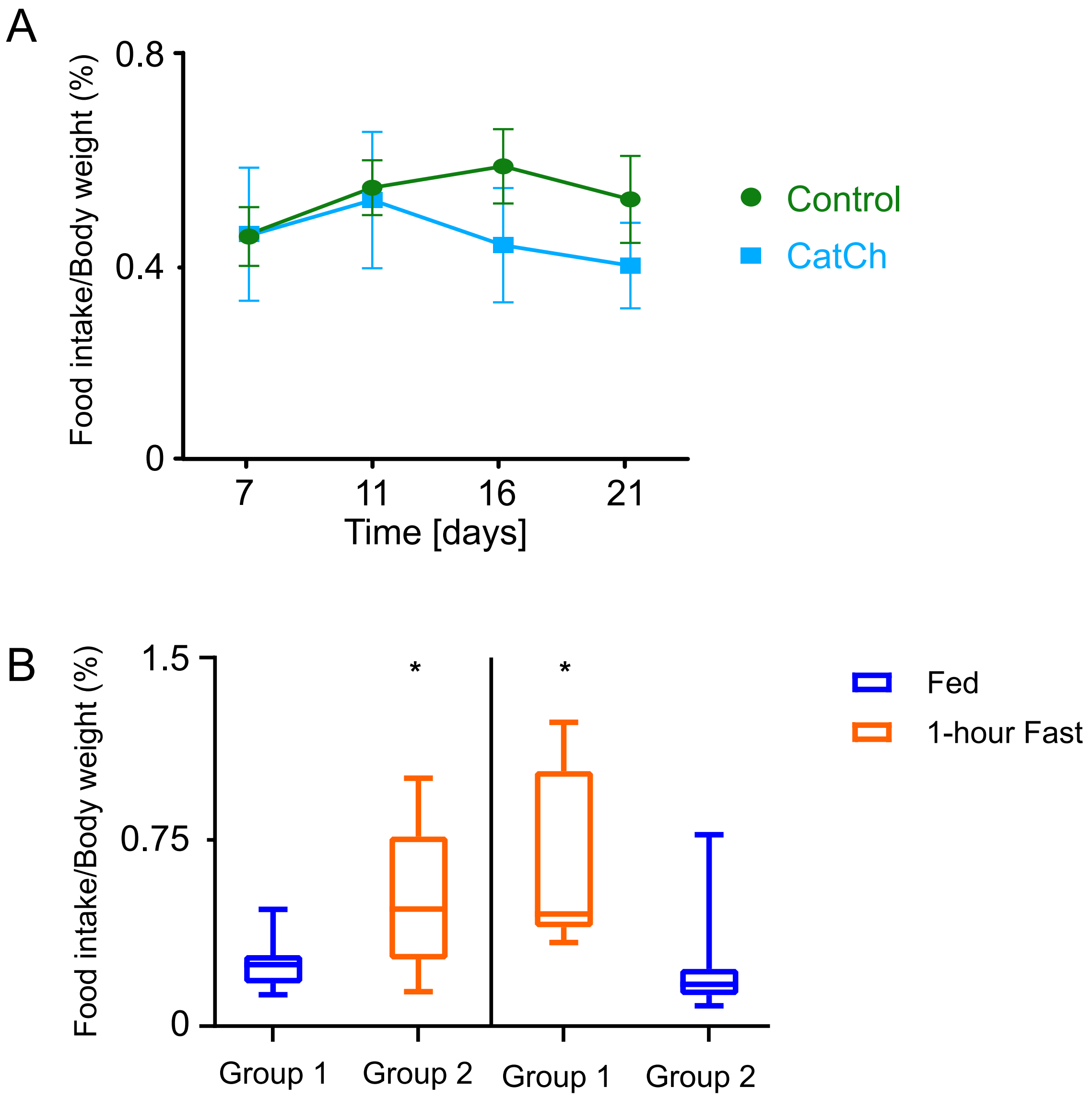


**Fig. S5. Food removal from homecage 1 hour prior to optostimulation increases food intake (when available) and occludes the behavioral effects of tanycytes optostimulation**:

(A) Time course of behavior response after activation of tanycytes by CatCh and controls. Food was removed 1 hour prior to the trial in the animals' homecage. No effect of the optostimulation was observed (2-way repeated measures ANOVA $p_{value} > 0.05$; n=8). Note that food intake was overall much higher than in Fig. 5B.

(B) 2 groups of naïve wild-type animals (n=8 each) were tested to confirm the effect of food removal on food intake. First week, group 1 was left for 45 minutes in presence of food and in the same condition as previously (time of the day, open-field, etc.) and food intake was recorded at the end. For group 2, food was removed from their homecage 1 hour prior to the test. Group 2 ate significantly more than group 1 (unpaired t-test $p_{value} = 0.03$).

The same groups were interchanged during the second week and now group 1 had food removed for 1 hour prior to the test whereas group 2 did not. Group 1 now consumed more food than group 2 (unpaired t-test $p_{value} = 0.01$).

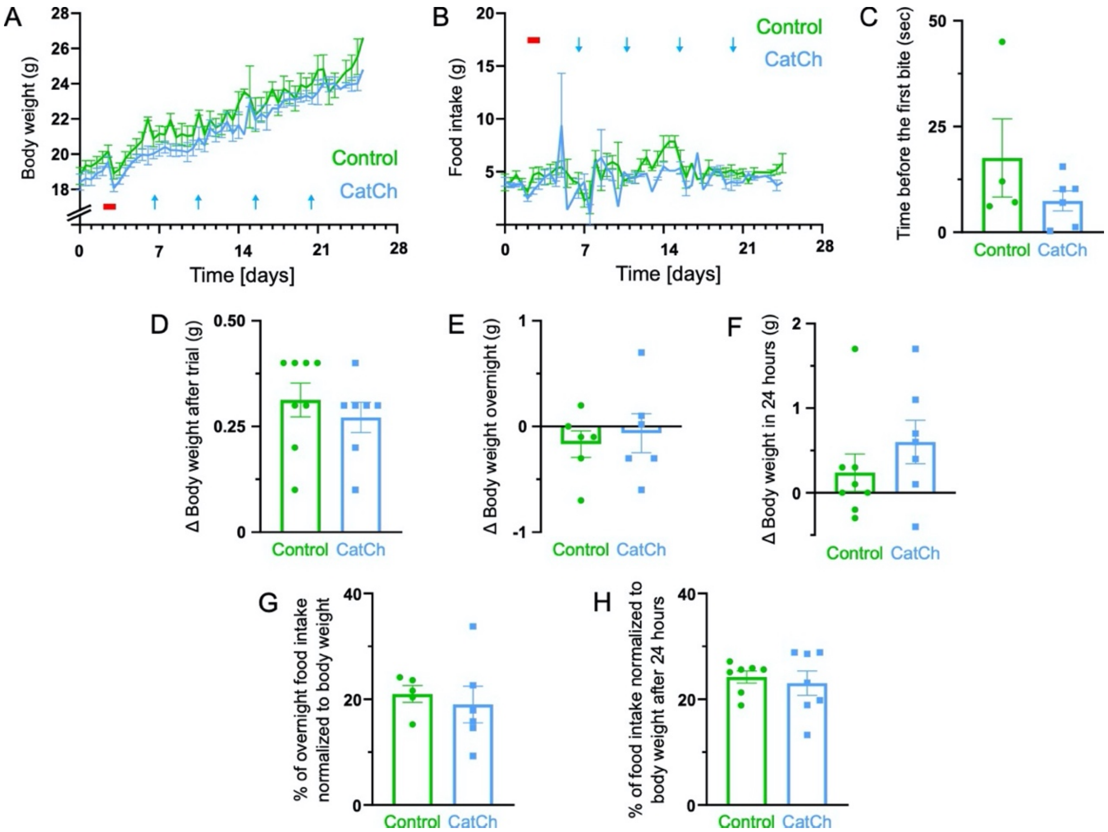


**Fig. S6. Optostimulation of tanycytes had no long-term effect on body weight or food intake**:

(A) Body weight time course for animals injected with adenoviruses (CatCh and control) over the 25 days. Surgery day was indicated by a red line and optostimulations by a blue arrow. No effect on body weight could be observed.

(B) Daily food intake was also recorded over the 25 days in the two groups and no effect was observed. Surgery day was indicated by a red line and optostimulations by a blue arrow.

(C) Time before animals had their first bite on pellet during trial test. No significant effect was observed between the groups (unpaired t-test $p_{value} = 0.2$).

(D) Body weight was recorded before and after the trial. Its variations did not show any significant changes between groups (unpaired t-test $p_{value} = 0.4$).

(E) Body weight was recorded before and overnight the trial. Its variations did not show any significant changes between groups (unpaired t-test $p_{value} = 0.6$).

(F) Body weight was recorded before and 24-hours after the trial. Its variations did not show any significant changes between groups (unpaired t-test $p_{value} = 0.3$).

(G) Percentage of food intake recorded overnight the trial. Food intake did not significantly change between groups (unpaired t-test $p_{value} = 0.6$).

(H) Percentage of food intake recorded for 24-hours after the trial. Food intake did not significantly change between groups (unpaired t-test $p_{value} = 0.6$).

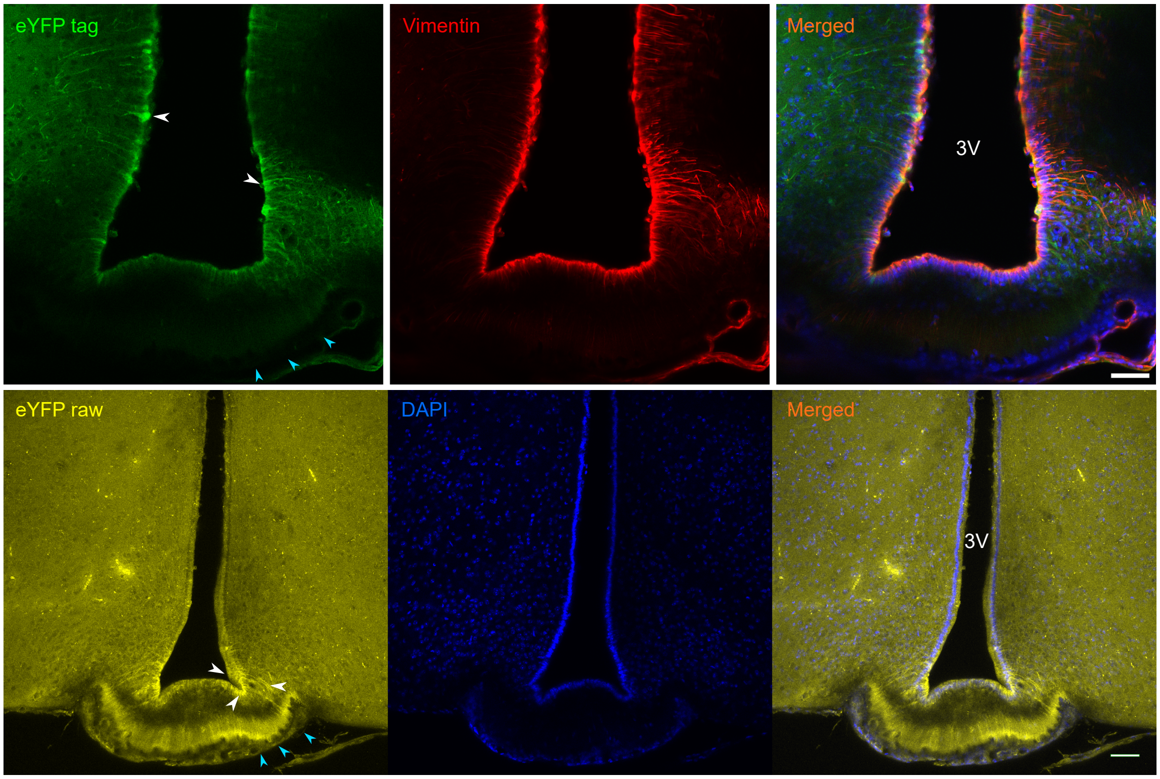


**Fig. S7. Viral expression in the hypothalamus**:

Top panel shows a wider field of view of Fig. 2 immunohistochemistry on hypothalamic sections.

Bottom panel shows a raw expression of the viral eYFP tag (no immunohistochemistry).

In both conditions no viral expression was observed in the *pars tuberalis* (blue arrows) or other parts of the hypothalamus. White arrows highlight tanycyte expression. Scale bars represent 50 µm.

| | NPY | POMC | Unknown neurons |
|---|---|---|---|
| **Plateau** | 1/5 | 1/3 | 2/10 |
| **Ramp depolarisation (mV)** | 13.4 ± 7.8 | 12.5 ± 6.3 | 12.8 ± 6.2 |
| **Latency to firing (sec)** | 95.5 ± 33.5 | 117.6 ± 77.6 | 177.7 ± 138.2 |

**Table S1. Heterogeneity in neuronal responses evoked by tanycytes**:

Table showing the differences in responses in between hypothalamic neuronal populations. No clear pattern could be observed.

**Movie S1.** $Ca^{2+}$ signaling evoked by stimulation of a single tanycytes (corresponds to Fig. 1 of main text).

**Movie S2.** $Ca^{2+}$ signaling in tanycytes and presumed astrocytes evoked by IR stimulation of a single tanycyte. This corresponds to SI Appendix, Fig. S1.

**Movie S3.** $Ca^{2+}$ signalling in tanycytes and a single presumed astrocyte evoked by IR stimulation of a single tanycyte.
