# Supplementary material for: Hypothalamic tanycytes generate acute hyperphagia through activation of the arcuate neuronal network
Source: Proc Natl Acad Sci U S A. 2020 Jun 8;117(25):14473–81. doi: 10.1073/pnas.1919887117 (PMC7322081; doi:10.1073/pnas.1919887117)
Supplement: Supplementary File [file pnas.1919887117.sd01.pdf]

Dataset

Figure 3 data:

No virus	AdV-Control	AdV-CatCh
1.70994281	1.97404946	16.6442565
1.33795713	-0.4702446	2.19441419
-0.5699989	0.6244662	15.3361985
10.7904002	1.4291424	13.0240847
-0.6486069	1.40439417	2.28323075
-1.3755224	0.93570635	5.47188402
-5.3194584		13.8543836
2.64352285		25.6259483
2.73617497		2.00919529
1.16275649		30.1867388
		1.74057667
		1.48863567
		1.23839887
		5.1822047
		6.74465542
		1.33695691
		11.2065652
		4.74395484
		2.59225603
		5.73992591
		8.10166492
		14.5794739
		3.46892898
		7.87000179
		4.66634199
		8.01419167
		3.66648989
		6.04559677
		9.08059559
		5.41164488

Figure 4C data:

Control	Before	At peak	After	Difference with peak
	-52	-49	-51	3
	-51	-49	-50	2
	-61	-62	-57	-1
	-65	-64	-61	1
	-54	-48	-50	6
	-50	-48	-48	2
	-41	-42	-41	-1
	-42	-43	-40	-1
	-53	-60	-65	-7
	-68	-69	-61	-1
UC				
	-62	-41	-61	21
	-55	-43	-54	12
	-70	-50	-70	20
	-61	-44	-61	17
	-64	-50	-66	14
	-54	-44	-56	10
	-77	-55	-80	22
	-40	-30	-43	10
	-65	-54	-71	11
	-48	-41	-45	7
NPY				
	-53	-37	-44	16
	-52	-36	-49	16
	-50	-29	-52	21
	-73	-53	-80	20
	-75	-47	-80	28
POMC				
	-48	-34	-44	14
	-64	-54	-71	10
	-56	-40	-52	16
100 nM MRS2500				
and 30 µM PPADS	-63	-66	-67	-3
	-54	-57	-68	-3
	-79	-77	-82	2
	-58	-60	-61	-2

	-68	-75	-72	-7
	-75	-74	-73	1
	-56	-61	-58	-5
	-75	-73	-78	2
	-53	-51	-56	2

Figure 4D data:

Time before firing	Pourcentage of Rhod Change before firing
141	7.8
191	6.7
145	23
190	10.8
160	4.1
160	9.2

Figure 5B data:

	7 days	11 days	16 days	21 days
AdV-Control				
18021	0.03	0.06	0.04	0.05
18022	0.03	0.03	0.01	0
18027	0.01	0.05	0	0.06
18028	0.02	0.01	0.09	0.03
18030	0.01	0	0	0
18032	0.01	0.04	0.04	0.08
18035	0.02	0.04	0.09	0.1
18036	0.01	0.01	0	0
AdV-CatCh				
18024	0.03	0.07	0.09	0.04
18025	0.02	0.05	0.11	0.06
18026	0	0.07	0.06	0.02
18029	0.02	0.05	0.1	0
18031	0.02	0.01	0	0.01
18033	0.02	0.02	0.07	0.07
18034	0.05	0.09	0.12	0

Figure 5C data:

AdV-Control	AdV-CatCh
0.43	0.18
0.55	0.04
0.27	0
0.49	0.44
0.05	0
0.31	0.19
0.55	0.38
	0

Figure 5D data:

AdV-Control	AdV-CatCh
0	0.34
0.48	0.13
0.04	0.13
0.09	0.46

0.17	0.31
0.14	
0.12	
0.14	

Figure 5E data:

Minutes	AdV-Control						
5	0	0	0	0	0	0	0
10	0	0	0	10200	0	0	0
15	0	0	0	0	3.047619	0	0
20	0	0	0	0	0	130200	16200
25	0	0	12000	0	5.054054	0	0
30	0	0	0	0	13200	0	0
35	171000	0.4642857	0	0	15.69444	76800	0
40	0	0	0	0	1.464789	0	0
Minutes	AdV-CatCh						
5	2400	0	18600	0	0	0	0
10	0	0	0	0	0	108000	0
15	0	6.759259	0	42600	0	0	40800
20	16200	0.3793103	0	41400	1800	65400	27000
25	21600	69600	16800	107400	0	0	0.5357143
30	73200	49200	14400	1.90625	0	104400	43800
35	0	177600	2.071429	14.04545	0	141000	27600
40	94200	0.1793103	0	0	0	0	48600

Figure S2 data:

Time	Distance	Speed
61.10974545	65.835	1.077324075
52.37887273	11.172	0.213292105
13.57741818	41.7825	3.077352369
54.26425455	31.477	0.580068781
275.2169818	48.881	0.177608953
25.20109091	15.7275	0.624080126
27.16054545	26.53	0.976784507
157.1421455	46.188	0.293924968
115.4305818	59.927	0.519160512
85.36301818	42.8555	0.502038247
90.79861818	34.425	0.379135726
32.8768	33.781	1.027502677
45.40043636	69.8555	1.538652612
17.22461818	33.2615	1.931044256
156.5500727	53.527	0.341916162
31.31370909	59.445	1.898369811
111.1518909	84.0095	0.755808105
27.11734545	32.934	1.214499408
14.59370909	10.5555	0.723291107
99.12825455	54.554	0.550337543
80.3428	79.798	0.993219056
4.6928	37.618	8.016109785
50.09098182	49.659	0.991376056
15.23552727	48.8675	3.207470219
12.10461818	30.9915	2.560303806
23.48461818	12.866	0.547847953
53.22825455	38.8355	0.729603109
42.27007273	48.6885	1.151843299
125.2317091	56.802	0.45357522
15.65807273	46.0135	2.938643906
68.88352727	61.12	0.887294865
230.1253455	65.6475	0.285268447
38.79716364	35.756	0.921613764
73.71847273	48.6855	0.660424697
118.3439636	14.873	0.125676034
16.49014545	39.5805	2.400251721
103.7934182	62.775	0.604807136
18.42912727	27.8175	1.509431217
8.728763636	50.35	5.768285418

24.24934545	49.855	2.055931781
36.86272727	26.854	0.728486522
204.6819273	18.5655	0.090704149
208.5620727	60.7865	0.291455197
30.06901818	65.425	2.175827611
129.8627273	76.276	0.587358679
56.21054545	65.145	1.158946235
21.31552727	50.553	2.371651395
12.52443636	24.535	1.958970391
7.828072727	76.807	9.811738173
173.7662545	66.486	0.382617443
64.18625455	69.656	1.085216773
70.44807273	28.24	0.400862634
75.13734545	69.362	0.923136153
4.694254545	41.244	8.786059554
82.96698182	41.112	0.495522425
101.7524364	56.776	0.557981725
109.5826182	23.668	0.215983159
101.7553455	57.836	0.568382916
175.3317091	76.699	0.43745082
62.61898182	52.434	0.837349929
64.18443636	59.309	0.924040209
32.87534545	80.24	2.440734809
6.262618182	58.215	9.295632962
12.52443636	36.044	2.877893979
40.70261818	56.147	1.379444432
51.6608	65.001	1.258226741
6.262618182	48.461	7.738137404
44.5828	56.015	1.256426245
3.873709091	22.689	5.857177054
3.873709091	42.149	10.88078609
3.131709091	55.262	17.64595574
76.70807273	79.423	1.03539298
70.44625455	26.166	0.371432096
6.262618182	43.179	6.894720187
76.70807273	41.4	0.539708515
79.83898182	51.004	0.638835802

Figure S4 data:

AdV-Control	AdV-CatCh
0.13043478	0.31531532
0.30567686	0.35555556
0.11764706	0.27906977
0.04237288	0.84745763

Figure S5A data:

	7 days	11 days	16 days	21 days
AdV-Control				
18088	0.612	0.707	0.504	0.319
18090	0.446	0.493	0.255	0.284
18092	0.222	0.734	0.358	0.162
18094	0.583	0.425	0.636	0.661
18096	0.153	0.597	0.923	0.825
18098	0.484	0.294	0.576	0.737
18100	0.486	0.415	0.653	0.69
18102	0.526	0.627	0.729	0.424
AdV-CatCh				
18089	0.966	0.547	0.211	0.394
18091	0.325	0.582	0.707	0.752
18093	0.98	0.617	0.761	0.588
18095	0.02	0.034	0.053	0.081
18097	0.026	1.079	0.031	0.051
18099	0.597	0.27	0.696	0.259
18101	0.363	0.936	0.709	0.489
18103	0.275	0.026	0.208	0.433

Figure S5B data:

Fed	1 h Fasting		1 h Fasting	Fed
I	II		I	II
0.2892562	0.829875519		0.39840637	0.25
0.47619048	0.560344828		0.34042553	0.08298755
0.24390244	0.24		0.49382716	0.11450382
0.28846154	0.598290598		0.97087379	0.16260163
0.25641026	0.140186916		1.2371134	0.1843318
0.18518519	1.00877193		1.05990783	0.77922078
0.17021277	0.366972477		0.42016807	0.17777778
0.12765957	0.3930131		0.41493776	0.16460905

Figure S6A data:

	Control									Catch						
	18021	18022	18027	18028	18030	18032	18035	18036		18024	18026	18029	18033	18034	18025	18031
Day1	18.1	19	20	17			20.1	18.4		18.4	17.8		19.8	19	17.1	
Day2	19.2	20.2			19.3	19.2	20	18.3		18.8		17.8	20	18.7		17.8
Day3			20	18	19.9	19.4	19.9	18.7			18.8	18.1	19.9	19	17.7	17.9
Day4	19.5	20	20.2	17.8	20	19	20.8	19		19.1	19	18.4	20.4	19.1	18.1	18.3
Day5	20.4	20.3	20.5	18.1	20.2	19.2	20.9	18.9		19.2	19.1	19	20.4	19	18.3	18.3
Day6	20.9	20.8	20.8	18.5	20.4	19.3	21.3	19.3		19.6	19.6	19.4	21.3	19.4	19	18.7
Day7	18.8	19.1	19.9	18	19.3	17.6	20.2	18.4		18.5	17.7	17.5	18.5	18.8	18	17.5
Day8	19.2	19.2	20	18.3	18.6	17.8	21	19.1		18.9	18.6	17.8	18	19.2	18.3	18.6
Day9	19.9	20.7	20.5	18.4	19.1	18.7	21.5	19.2		20	19.2	18.6	18.4	19.7	18.4	18.7
Day10	20.8	21	20.7	18.7	19.5	19.4	21.6	19.4		19.8	19.8	19.4	19.9	19.6	18.6	19
Day11	21.3	21.5	21.2	18.6	20	19.3	22.1	19.5		20	20.2	19.4	20.6	20	18.8	19.3
Day12	21.6	22.1	21.6	18.8	19.9	19.6	22.5	20.1		20.2	20.5	19.4	21.3	20.3	18.6	19.5
Day13	21.9	22.1								20.2	20.7				18.9	
Day14	21.4	22.3	22.1	19.3	20.1	19.7	22.3	19.8		20	20.6	19.4	21.2	20.4	19	19.6
Day15	21.7	22.8	22.3	19.7	20.2	19.8	22.4	20.3		20	20.8	19.5	21.8	20.7	19.5	19.3
Day16	21.7	22.4	22.4	19.8	20	19.8	22.6	20.2		20.4	20.8	19.6	22	20.9	19.4	19.7
Day17	21.6	22.3								20.3	20.8				19.6	
Day18	21.3	22.4	22.4	19.9	19.7	19.4	22.6	20		20.2	21.2	19.3	21.5	20.8	19.5	19.1
Day19	21.9	22.7	22.4	19.4			21.2	19.2		20.5	21.2		19.9	19.9	19.5	
Day20	21.7	22	22.6	19.9	19.8	18.2	23.1	20.9		20.3	21.6	18.2	21.3	21.4	19.7	18.3
Day21	22.2	22.6	21.2	18.5	20.2	20	23	20.9		20.6	20.5	19.9	21.8	21.6	18.5	19.5
Day22	22.2	22.5								20.9	22				19.8	

Day23	20.9	22.4	22.8	20.4	20	20				20.9	21.7	20.3			19.9	19.7
Day24	21	22.4	23.2	20.8			23.5	20.7		20.7	22		22.6	22	20.1	
Day25	21.2	22.3			20.3	20.8	23.7	20.8		20.7		20.6	22.3	22.2		20
Day26			23.4	20.7	20.7	20.7	23.6	21			21.8	20.9	22.7	22.1	20.3	20.3
Day27	22.4	22.5								21.3	22.4					
Day28	22.9	22.5	24	20.7	20.7	20.8				21.1	22.4	20.9				20.3
Day29	23.9	23.1	23.6	21						20.9	22.4					
Day30	23.7	23.4								21.1						
Day31							25	21.5					23.2	22.7		
Day32					21.2	21.4	25.1	21.3				21.5	23.1	22.3		20.8
Day33			24.3	21.2	21.6	21.5	25	21.7			22.7	21.9	23.6	22.5		20.7
Day34	24.5	23.7	24.6	21.2	21.3	21.6	25.1	21.6		22.4	22.6	22.1	24	22.6		18.8
Day35	24.1	23.3								22.6						
Day36	24.6	23.1	25.4	21.7	21.4	21.3				22.5	23	22.3				
Day37	25.2	23.3	25	21.4			25.4	22		22.7	22.6		24.3	22.7		
Day38	25.2	24.1			22.9	21.4	25.2	22.3		22.8		22.6	24.2	22.9		
Day39			25	21.8	22.9	21.7	25.6	22			22.6	22.8	24.2	22.8		
Day40	25.8	24.8	25.8	21.9	23.3	21.8	26	22		23.4	22.7	23	24	22.8		
Day41	25.4	24.9	24.8	22.3	23	21.9	25.7	22.4		23.5	22.8	22.6	24.3	23.4		
Day42	25.4	25	25	22.1	24.1	22.5				23.8	22.8	22.9				
Day43	25.9	25.3	25.6	22.3						24.2	22.8					
Day44	25.8	25.4					25.7	22.4		24.1			24.4	23.6		
Day45					24.3	22.2	25.1	22.5				23.5	24.8	23.6		
Day46			25.9	22.4	24.8	22.4	25.6	22.3			23.7		24.7	23.7		
Day47												23.7				
Day48	26.6	25.9	26.4	22.3	24.7	22.9	26.1	22.6		24.3	23.6	24.1	24.1	23.5		

Day49	26.6	25.9	26.2	22.6	25.4	22.7				24.7	23.4	23.8				
Day50	26.7	26.5	26.4	22.5						24.4	23.6					
Day51	26.6									24.8						

Figure S6B data:

	Control									Catch						
	18021	18022	18027	18028	18030	18032	18035	18036		18024	18026	18029	18033	18034	18025	18031
Day1	5.3	5.1					3.7	1.9		4.9	4.65		3.2	3.5		
Day2	5.65	4.75			3.4	3.4	4.2	2.7		3.95	4.65	2.9	3.7	3.9		3.3
Day3	5.65	4.75	5.8	3.6	5.5	3.1	4.4	5		3.95	3.5	4.2	3.8	4.1	3	3.6
Day4	2.3	4.3	6.3	4.4	4.8	3.5	3.8	1.3		4.1	3.2	4.5	3.7	3.5	4.4	3.8
Day5	3.1	3.9	2.3	4.9	2.6	2.2	2.6	3.4		3	2.1	1.4	2.6	2.1	3.5	2.5
Day6	3.25	5.3	8.4	3.4	4	3.7	2.9	6.6		2.4	2.1	2.5	4.5	3.7	3.4	3.9
Day7	3.25	4.9	7.9	3.5	5.8	4.6	5.7	3.6		5	2.7	3.3	1.8	4.4	5.3	4.1
Day8	4.8	7.2	0.5	4.5	5.1	5	4.6			4.8	6.2	6.1	1.2	5.2	4.8	3
Day9	9.4	7.3	3.8	4.2	5.4	0.8	4	3.4		5.2	3.8	4.5	6.9	4	3.5	6.1
Day10	6.6	7.5	3.5	3.6	4.2	5.1		6.6		4.3	2.9	3.9			4.3	4
Day11	4.8	6.5	7.1	4.9			4			5.4	3.9		6	3.3	3.9	33.9
Day12	3.5	6.2								1.4						
Day13	3.6	4.2						4.6		2.4						
Day14	3.6	9.3	5.1	5	4.2	3.1	0	1.2		4	4.1	4.7	0	0	6.2	5
Day15	2.7	2.3	4.3	4.1	0	0	2.7			5.7	3.7	4.6	6	3.6	4.7	0
Day16	4.2	6.1	0	0						1.9	0					
Day17	4.6	6.9						7.1		5.8						
Day18	5.2	1.2	5.8	3.2			7.4	2.3			1.4		4.1	6	13.8	

Day19	5.8		9.4	5.9			3.1	4		2.7	9.1		5.6	4	5.4	
Day20	5.9	8.7	6	7.2	5.3	4.7	3.7			5.6	4.7	4	2.1	4.2	6	6.2
Day21	3.4	7.8								1.7						
Day22	3.7	4.6								5						
Day23	3.4	5.2	4.8	4.2				4.5		4.3	5.1				3.7	
Day24	4.2	5.7					3.6			3.5	4.85		5.7	3.7		
Day25	5.7	7.05			6.1	3.4	5.9			4.8	4.85	5	4.3	4.2		5
Day26	5.7	7.05								4.8						
Day27	5.4	5.1								3.1						
Day28	6.9	7.9	5.2	5.1						4.2	4.5					
Day29	6	8.4								5.9	5.1					
Day30	7.3	8.4								5.1	5.1					
Day31	7.3	8.4								5.1	5.1					
Day32	7.3	8.4					4.1	4		5.1	5.1		7	4.2		
Day33	3.3	8.4			2.7	4.5	3.5			5.1	4.2	4.4	2.6	3.8		0.4
Day34		5.8								3.7						
Day35		4.3								6.8						
Day36	2.8	4.4	4.3	5.6				4.8		3.3						
Day37	5.8	7.2					4.4	4.3		3.4			5	4.1		
Day38					4.4	4.6	5.4	5.6				5.4	5.5	4.6		
Day39			4.4	4.6	4.5	6.1	5.9	4.1			3.8	5.5	5.6	5.7		
Day40	4	6.4	3.6	6.5	5.4	4.2	4.5			5.2	4.2	2.9	2.8	4.1		
Day41	4.4	7.3	5.9	5.1	4.2	4				4.6	5.1	2.4				
Day42	4.1	4.2	5.7	5.1						3.7	4.2					
Day43	4.3	5.6						4.9		4						
Day44							3.9	4.3					5	4.6		

Day45					5.8	4.8	4.3						4.6	4.2		
Day46																
Day47												4.6				
Day48	5.6	5.5	3.9	4.7	5	4.8				4.9	3.9	4.2				
Day49	5.2	6.4	5.2	4.1						3.2	4.2					
Day50	4.9	6.7								4						

Figure S6C data:

Control	Catch
12	0.3
45	1.24
7.1	10.25
6.13	10.17
	7
	15.5

Figure S6D data:

Control	Catch
0.3	0.3
0.4	0.3
0.1	0.3
0.4	0.1
0.3	0.2
0.4	0.3
0.4	0.4
0.2	

Figure S6E data:

Control	Catch
0	-0.3
-0.7	0.02
-0.3	-0.3
-0.1	-0.6
-0.1	0.7
0.2	0.1

Figure S6F data:

Control	Catch
0.3	0.1
0	0.6
-0.3	-0.4
-0.2	1.7
0.1	1.1
1.7	0.7
0	0.4
0.3	

Figure S6G data:

Control	Catch
20.3703704	22.6600985
15.2466368	15.8163265
21.6080402	14.5728643

23.6180905	9.27835052
24.137931	33.7837838
	17.9245283

Figure S6H data:

Control	Catch
21.2962963	28.5714286
18.8340807	13.2653061
27.1493213	28.8461538
25.8883249	23.1155779
25.1256281	28.8659794
25.6281407	18.9189189
25.6157635	19.8113208
